# Associations of Environmental Conditions and *Vibrio parahaemolyticus* Genetic Markers in Washington State Pacific Oysters

**DOI:** 10.3389/fmicb.2019.02797

**Published:** 2019-12-04

**Authors:** Aspen Flynn, Benjamin J. K. Davis, Erika Atherly, Gina Olson, John C. Bowers, Angelo DePaola, Frank C. Curriero

**Affiliations:** ^1^Spatial Science for Public Health Center, Bloomberg School of Public Health, Johns Hopkins University, Baltimore, MD, United States; ^2^Department of Epidemiology, Bloomberg School of Public Health, Johns Hopkins University, Baltimore, MD, United States; ^3^Office of Environmental Health and Safety, Division of Environmental Public Health, Washington State Department of Health, Olympia, WA, United States; ^4^Public Health Laboratories, Division of Disease Control and Health Statistics, Washington State Department of Health, Shoreline, WA, United States; ^5^Office of Analytics and Outreach, Center for Food Safety and Applied Nutrition, Food and Drug Administration, College Park, MD, United States; ^6^Angelo DePaola Consulting, Coden, AL, United States

**Keywords:** *Vibrio parahaemolyticus*, *Crassostrea gigas*, Pacific oysters, Washington, temperature, genetic markers, seafood-borne illness

## Abstract

*Vibrio parahaemolyticus* is a naturally occurring bacterium in estuarine waters and is a major cause of seafood-borne illness. The bacterium has been consistently identified in Pacific Northwest waters and elevated illness rates of vibriosis in Washington State have raised concerns among growers, risk managers, and consumers of Pacific oysters (*Crassostrea gigas*). In order to better understand pre-harvest variation of *V. parahaemolyticus* in the region, abundance of total and potentially pathogenic strains of the bacterium in a large number of Washington State Pacific oyster samples were compared with environmental conditions at the time of sampling. The Washington Department of Health regularly sampled oysters between June and September at over 21 locations from 2014 to 2018, resulting in over 946 samples. *V. parahaemolyticus* strains carrying three genetic markers, *tlh*, *trh*, and *tdh*, were enumerated in oyster tissue using a most probable number-PCR analysis. Tobit regressions and seemingly unrelated estimations were used to formally assess relationships between environmental measures and genetic markers. All genetic markers were found to be positively associated with temperature, independent of the abundance of other genetic markers. Surface water temperature displayed a non-linear relationship, with no association observed between any genetic marker in the warmest waters. There were also stark differences between surface and shore water temperature models. Salinity was not found to be substantially associated with any of the genetic variables. The relative abundance of *tdh*+ strains given total *V. parahaemolyticus* abundance (pathogenic ratio *tdh*:*tlh*) was negatively associated with water temperature in colder waters and decreased exponentially as total *V. parahaemolyticus* abundance increased. Strains carrying the *trh* gene had a pronounced positive association with strains carrying the *tdh* gene but was also negatively associated with the *tdh*:*tlh* pathogenic ratio. These results suggest that there are ecological relationships of competition, growth, and survival for *V. parahaemolyticus* strains in the oyster tissue matrix. This work also improves the overall understanding of environmental associations with *V. parahaemolyticus* in Washington State Pacific oysters, laying the groundwork for future risk mitigation efforts in the region.

## Introduction

*Vibrio parahaemolyticus* is a Gram-negative, halophilic bacterium naturally present in many coastal waters throughout the world (CDC, 1998; Ansaruzzaman et al., 2005; Su and Liu, 2007; Kirs et al., 2011; Velazquez-Roman et al., 2014; Wu et al., 2014; Martinez-Urtaza et al., 2016). It is the leading cause of seafood-borne bacterial gastroenteritis (Scallan et al., 2011). While symptoms are often mild and self-limiting, *V. parahaemolyticus* infections (i.e., vibriosis) can at times lead to life-threatening septicemia. The most common cause of vibriosis in humans is the consumption of raw or undercooked seafood (Su and Liu, 2007). The bacterium can accumulate in marine organisms, especially in shellfish due to their filter-feeding behavior (DePaola et al., 2003).

The presence of *V. parahaemolyticus* in the environment and its capacity for human infection is widespread. The first recorded outbreak occurred in Osaka, Japan, in 1950 (Fujino et al., 1953), and since 1996, there have been more frequent vibriosis outbreaks in North America, South America, Western and Eastern Europe, and Asia (CDC, 1998; Velazquez-Roman et al., 2014; Wu et al., 2014; Gonzalez-Escalona et al., 2016; Haendiges et al., 2016; Martinez-Urtaza et al., 2016). The Pacific Northwest (PNW) region of the United States is an environment in which pathogenic *V. parahaemolyticus* strains are relatively abundant. A large outbreak of cases first occurred in this region in 1997, when 209 people became ill from eating shellfish harvested in PNW and one person died (CDC, 1998). Since that time, there have been additional outbreaks and increased incidence of sporadic cases in the region, particularly in Washington State where the majority of shellfish are harvested (McLaughlin et al., 2005; National Marine Fisheries Service et al., 2017; Washington Disease Control and Health Statistics, 2018). Cases have continued to rise over time despite multiple efforts by the Washington State Department of Health (WDOH) and shellfish growers to restrict harvesting by time and place and to implement austere post-harvest controls (Paranjpye et al., 2012; Washington State Board of Health, 2015).

While all *V. parahaemolyticus* bacteria possess a thermolabile hemolysin gene (*tlh*), not all strains can cause vibriosis. Common indicators of pathogenicity among *V. parahaemolyticus* bacteria are the thermostable direct hemolysin and the thermostable direct-related hemolysin genes (*tdh* and *trh*) (Shirai et al., 1990; Panicker et al., 2004). While neither of these genes are required or consistently predict pathogenicity, they have been shown to be regularly associated with type III secretion systems that lead to human infection, and so are frequently used to measure virulence factors in environmental isolates (Paranjpye et al., 2012; Zhang and Orth, 2013). It is worth noting that type VI secretion systems, while not associated with the hemolysin genes, also appear to play an important role in the pathogenicity of some strains (Salomon et al., 2013; Raghunath, 2014). PNW waters and oysters have relatively high levels of *tdh* and *trh* genetic material compared to other North American bodies of water, with the bacterium population being comprised of a wide array of strains that participate in regular genetic recombination (Hazen et al., 2010; Paranjpye et al., 2012; Turner et al., 2013). Many of the bacterium strains common to PNW waters, particularly those that are *tdh*+ /*trh*+, have also been frequently associated with vibriosis cases in Washington and throughout the continent (Martinez-Urtaza et al., 2013; Turner et al., 2013; Velazquez-Roman et al., 2014; Xu et al., 2017). It should be noted that observed *trh* levels in PNW may be due in part to the presence of *Vibrio alginolyticus* bacteria strains, calling into question the utility of the marker (Gonzalez-Escalona et al., 2008).

There has been extensive research on the range of environmental conditions that are associated with the presence and abundance of *V. parahaemolyticus* (DePaola et al., 2003; Turner et al., 2014; Johnson, 2015; Paranjpye et al., 2015; Davis et al., 2017). There is a well-documented positive correlation between water temperature and *V. parahaemolyticus* abundance, and consequently water temperature is used as the primary environmental input in the U.S Food and Drug Administration’s risk assessment for *V. parahaemolyticus* in oysters (USFDA, 2005). The bacteria cannot survive in freshwater, but concentrations at higher salinity levels vary widely by other water column conditions. Water clarity has been negatively associated with *V. parahaemolyticus* abundance, potentially due to the surface area suspended particles provide for the bacteria to adhere and grow. High levels of plankton and low levels of dissolved oxygen have also been found to be associated with greater bacterium abundance. Unfortunately, many of these associations vary significantly across geographic regions, time periods, and by the genetic marker of interest, making it difficult to identify broadly applicable relationships between environmental determinants and abundance of the bacterium (DePaola et al., 2003; USFDA, 2005; Turner et al., 2013; Johnson, 2015; Paranjpye et al., 2015; Davis et al., 2017). A notable example of such variation is the reported negative associations between the relative abundance of *tdh*+ strains in Gulf Coast oysters with both water temperature and the total abundance of *V. parahaemolyticus* (DePaola et al., 2003). Some of the variation in environmental associations may be contextualized as additional datasets of *V. parahaemolyticus* abundance are collected in pre-harvest settings that encompass large periods of time and geographic areas.

Given the health concerns related to *V. parahaemolyticus* in Washington, expected increases in illness rates as higher latitude waters continue to warm over time (Baker-Austin et al., 2017), and the economic impact to one of the largest groups of oyster producers in the country, further investigation into the environmental determinants of the bacterium in the region is warranted. The current study utilizes a large dataset of regularly monitored harvesting sites and public beaches in Washington State. The dataset contains one of the largest collections of oyster samples ever analyzed for *V. parahaemolyticus* and spans a substantial range of locations and time periods. The size and extent of the current dataset further allows for investigation into environmental associations independent of the concentrations of other genetic markers as well as statistical interactions between determinants. Overall, this analysis evaluated the relationships between absolute and relative abundance of *V. parahaemolyticus* strains carrying selected genetic markers and water temperature and salinity in Washington State.

## Materials and Methods

Since 2006, WDOH has been regularly sampling Pacific oysters (*Crassostrea gigas*) to measure *V. parahaemolyticus* abundance. Each year, intertidal shellfish harvesting beds were sampled as frequently as every week from June to September, primarily in the Hood Canal and South Puget Sound but also less frequently in the coastal bays and northern shellfish harvesting waters resulting in over 200 samples per year. For each sample, 13–18 live oysters approximately 10 cm in length were collected, when possible, after being exposed to the air by the receding tide. Oysters were rinsed in seawater before being sealed in a bag and then chilled with ice in an insulated container. Shoreline water and ambient air temperature were recorded with a thermometer. Surface water temperature was also measured where the total water depth was at least 0.6 m. An additional oyster was shucked and its tissue temperature recorded before being discarded. A refractometer was used to measure salinity of the surface water.

Oyster samples were analyzed by the WDOH Public Health Laboratory for *V. parahaemolyticus* abundance using a previously described most-probable-number (MPN)-based TaqMan real-time PCR assay (Glover, 2015). Three genetic markers were utilized: the species-specific thermolabile hemolysin gene (*tlh*), the virulent thermostable direct hemolysin gene (*tdh*), and the thermostable direct-related hemolysin gene (*trh*). Upon receipt at the laboratory, an oyster from each sample was immediately shucked and the internal temperature was recorded to ensure proper temperature control during transit. Oysters were stored at 2–8°C until sample processing began. All shells were cleaned and shucked following guidelines set by the American Public Health Association (APHA, 1970). At least 12 oysters per sample were washed and all debris removed prior to aseptically shucking from the shell (instead of the hinge) in order to prevent contamination of the oyster tissue. Oyster tissue was then homogenized and inoculated into a MPN dilution series, all within 24 h of initial sample collection. The MPN portion of the assay was conducted using a three-tube six decimal dilution series ranging from 1 to 0.00001 g of oyster tissue enriched in alkaline peptone water (APW) to measure concentrations of the targets (Blodgett, 2017). Samples were incubated for 18–24 h at 35°C. Once removed from the incubator, tubes were read for turbidity and lysed within 1 h.

The real-time PCR assay was used as a qualitative determination of the absence or presence of each targeted gene in the individual tubes of the MPN dilution series. DNA isolation was performed on the Roche MagNA Pure LC instrument using the MagNA Pure LC DNA Isolation Kit III (Roche, Indianapolis, IN, United States). Real-time PCR analysis was performed on the Life Technologies ViiA7 real-time PCR instrument (Thermo Fisher Scientific, Waltham, MA, United States) using a 384-well platform. Commercially prepared mastermix was utilized in the assay (Bioline SensiFAST^TM^ Probe Hi-ROX Kit BIO-82020, Meridian Bioscience, Cincinnati, OH, United States) with the following cycling parameters: an initial denaturation of template at 95°C for 10 min, followed by 45 cycles of denaturation at 95°C for 15 s, and combined annealing and extension at 59°C for 60 s. An artificially created internal control (IC) plasmid was added to the PCR mastermix in order to detect any inhibitors within each sample. In addition to the IC plasmid, extraction controls were included on every Magna Pure cartridge and run on the PCR assay. Amplification controls were also included on every real-time PCR run. The positive extraction control was *V. parahaemolyticus* ATCC 49398^TM^ and the positive amplification control was a combination of previously extracted DNA from *V. parahaemolyticus* ATCC BAA-240^TM^ and from a *trh*+ Washington State clinical isolate. The negative extraction and amplification controls were both sterile molecular grade water. If PCR results did not demonstrate expected values for all controls, samples were reprocessed. There have been no reported instances by the WDOH Public Health Laboratory of an incorrect control value not being resolved during reprocessing.

Due to the artificial enrichment of the bacteria in APW, the real-time PCR resulted in positive tubes displaying consistent amplification across all dilutions of the MPN. When late amplification occurred (after 40 cycles), the PCR was repeated in duplicate to ensure appropriate reporting. In instances where *trh* was detected in the absence of *tlh*, indicating the presence of only *V. alginolyticus* (Gonzalez-Escalona et al., 2006), *trh* values were not reported. The positive and negative results generated on the PCR assay were applied back to the MPN dilution series in order to enumerate the amount of each genetic marker present in the oyster sample. The limit of detection for all genetic markers from the MPN assay was 0.3 MPN/g and the upper limit of quantification was 110,000 MPN/g.

**FIGURE 1:**
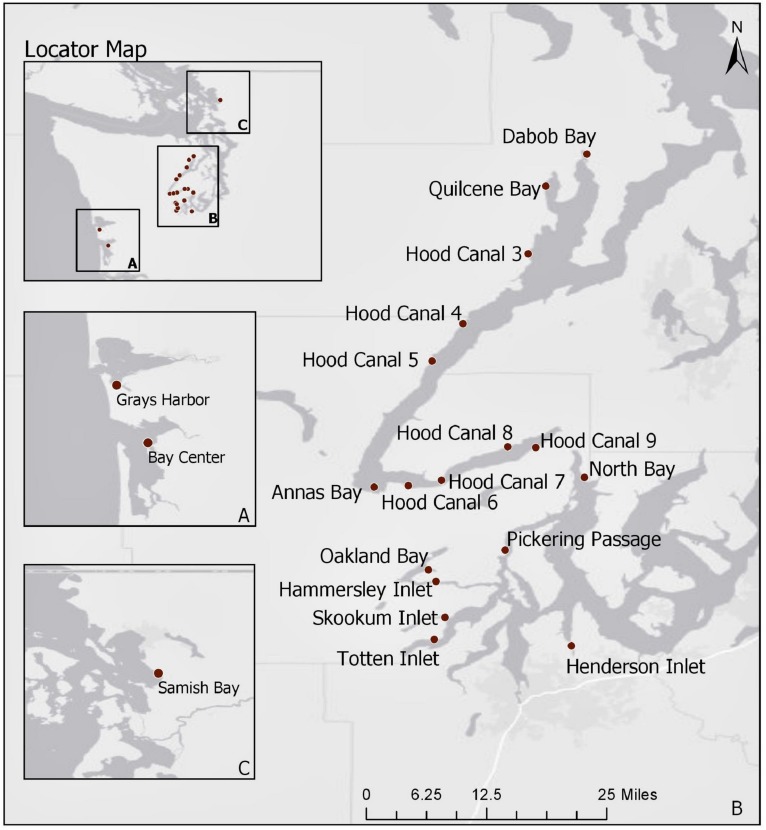
Map of Washington State shellfish sampling sites between 2014 and 2018. Panel **A** displays the coastal bays, panel **B** (main map) includes South Puget Sound and Hood Canal, and panel **C** displays Samish Bay in the northern waters. Basemap attributed to ESRI (2019b).

Current statistical analyses are limited to 2014–2018 samples as the *trh* genetic marker was not targeted before this time period. A total of 1,025 oyster samples were tested for *V. parahaemolyticus* at 25 sites during these years. In order to limit analysis to well-sampled locations, observations were excluded from the current analysis if taken from sites that were sampled on less than 12 occurrences throughout the 5-year period, or if only one sample was taken at a site for a given year. Furthermore, records that had missing information for any of the environmental or genetic variables were removed. This reduced the current analysis to 879 samples taken at 21 sampling sites (Figure 1). Note that only one sampling site from the northern waters, Samish Bay, was included in the analysis after the reduction in sample size.

An additional genetic variable, a *tdh*:*tlh* pathogenic ratio, was created to assess the relative abundance of tdh+ strains for each oyster sample collected. All genetic variables were positively skewed and non-negative and so were log-transformed for all statistical analyses. Natural logs were used when variables were the outcome of interest in regression analysis, but in all other instances, log_10_-transformations were used to facilitate interpretation.

Exploratory analyses included developing boxplots and histograms of each environmental and genetic variable and scatter plots to compare bivariate relationships. Non-linear trends were visually identified using non-parametric local regressions (LOESSs). Pearson correlation were used to quantify the relationships between all genetic and environmental variables. For exploratory analyses, and when used as explanatory variables in regression models, censored genetic values were imputed: those below the limit of detection were halved and those above the upper limit of quantification were doubled.

Tobit regression models were used to characterize the linear associations between environmental measures and *V. parahaemolyticus* abundance while accounting for left- and right-censored genetic data (limit of detection and limit of quantification, respectively). Separate models were developed for each genetic marker. For the pathogenic ratio, a more sophisticated regression analysis was undertaken in order to preserve the individual censored data for both the numerator (*tdh*) and denominator (*tlh*). Zellner’s seemingly unrelated regression (SUR) model was first performed simultaneously on linear *tdh* and *tlh* regressions using only non-censored data; residuals were evaluated using a Pearson’s correlation along with the Breusch–Pagan test of independence (Zellner, 1962, 1963; Breusch and Pagan, 1980). If residuals were found to be independent, Tobit regressions for *tdh* and for *tlh* could be estimated simultaneously to obtain the joint parameter vector and variance-covariance matrix for coefficients by using a seemingly unrelated estimation technique (Weesie, 1999). Given that all genetic variables were log-transformed, these estimations could be used to calculate the *tdh*:*tlh* ratio’s regression parameters and standard errors by subtracting the two sets of estimations via linear combination. The results of all genetic marker models are reported in their exponentiated form, and as such can be interpreted as the relative change in the geometric mean of gene abundance.

For each genetic marker, univariate models were compared to multivariate models that included year, region, and all environmental covariates (Multivariate 1), or that further included all non-redundant genetic covariates (Multivariate 2). Year and region were both treated as categorical variables, and as such indicator variables were included in each multivariate model. Multivariate 1 models were used to determine whether any environmental associations were redundant or unique to certain locations and time periods. Multivariate 2 models were used to determine if environmental associations were mediated by the abundance of total *V. parahaemolyticus* or potentially pathogenic strains. Note that a Multivariate 2 model was not created for regressions with *tlh* as the outcome, as potentially pathogenic strains make up a part of the total bacterium abundance. Potential interactions within and between environmental and genetic variables were formally tested by adding them to the regression models. When interactions were deemed to be statistically significant (α = 0.05), linear combinations of the original variable term and the interaction term were used to quantify the impact and statistical significance of the effect modification.

Given the potential redundancy of surface water temperature with shore water temperature, multicollinearity was assessed using the variance inflation factor on sensitivity regression analyses. Model fit of the two covariates was also assessed using the Akaike information criterion (AIC). An additional sensitivity analysis was conducted by removing Henderson Inlet observations, as the sampling site is connected to a freshwater inlet and had substantially lower salinity levels than other locations.

All statistical analyses were performed in Stata 13 (StataCorp, 2011). Maps were created in ArcGIS Pro version 2.3.2 and additional plots were created in R statistical software version 3.5.2 using the ggplot package (Wickham, 2009; R Core Team, 2018; ESRI, 2019a).

## Results

The map of Washington State shellfish sampling sites is shown in Figure 1. The mean number of samples taken at each site across the 5-year sampling period was 50.5 with a range of 14–72. The number of samples across years was roughly similar for each site. Total ranges of environmental variables are as follows: ambient air temperature (5.2, 35.0°C); surface water temperature (12.5, 35.4°C); tissue temperature (7.7, 38.4°C); salinity (3.0, 35.0‰). All genetic variables had values below the limit of detection (*N_*tlh*_* = 9, *N_*trh*_* = 101, *N_*tdh*_* = 469). Only *tlh* had values above the upper limit of quantification (*N* = 4) while *trh* had a maximum observable value of 46,000 MPN/g and *tdh* a value of 430 MPN/g. The *tdh*:*tlh* ratio ranged from 0 to 100%. Using a stratified random sampling approach across all monitoring years (*n* = 150 for each target), the proportion of oyster samples that experienced late amplification in duplicate PCR runs was estimated to be 2.0% (95% CI: [0.7, 3.3%]) occurring in eight samples for the *tlh* target and one sample for the *trh* target. Late amplification increased abundance on average by a factor of 3.4 MPN/g (95% CI: [1.6, 7.0 MPN/g]).

**FIGURE 2:**
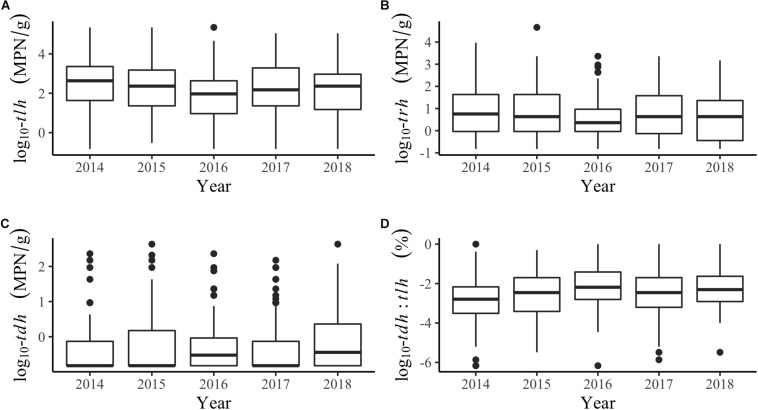
Boxplots of genetic markers by year for **(A)** *tlh*, **(B)** *trh*, **(C)** *tdh*, and **(D)** the *tdh*:*tlh* pathogenic ratio.

Additional descriptions of the environmental and genetic variables can be found in Table 1 and Figure 2. All temperatures showed similar trends across years, although tissue temperature frequently reached higher values in the Hood Canal and South Puget Sound relative to Samish Bay and the coastal bays. Salinity showed no noticeable variation across region or year. *tlh* values were slightly lower in 2016 and seemed to be noticeably higher in Hood Canal and lower in Samish and the coastal bays. *trh* abundance was over an order of magnitude lower than *tlh* overall and was particularly low in Samish Bay. When *tdh* was observable, abundance was usually under 1 MPN/g and no samples in Samish Bay had detectable levels of *tdh*. The pathogenic marker also appeared to be slightly higher in 2016 and 2018. The coastal bays appeared to have overall much higher *tdh*:*tlh* values than other sampling regions. Salinity appeared negatively skewed, likely due to the lower salinity measurements in Dabob Bay, Henderson Inlet, and Hood Canal 3 and 8 (results not shown). Log-transformed *tdh* appeared positively skewed, and this pattern persisted across sampling location and year.

**TABLE 1:** Descriptive characteristics of environmental and genetic variables, as well as their variation across sampling regions.

	**Overall**	**Region**			
		**Hood Canal**	**South Puget Sound**	**Coastal Bays**	**Samish Bay**
	***N* = 879**	***N* = 468**	***N* = 381**	***N* = 39**	***N* = 48**
		
**Characteristic**	**Median (IQR)**	**Median (IQR)**			
Ambient air temperature (°C)	18.4(15.7,21.7)	18.2(15.7,21.8)	19.0(16.0,22.0)	17.2(15.9,18.6)	18.0(15.7,19.1)
Surface water temperature (°C)	19.0(17.4,21.0)	19.4(17.3,21.4)	18.8(18.0,20.6)	18.5(17.5,19.2)	20.4(16.5,23.6)
Tissue temperature (°C)	21.6(18.0,26.0)	21.7(17.7,26.3)	22.0(18.4,26.2)	19.3(17.1,21.0)	20.4(16.7,23.0)
Salinity (‰)	26.7(24.3,28.5)	25.2(21.4,27.0)	27.3(26.0,29.0)	28.0(26.0,29.5)	28.7(26.3,29.8)
*tlh* (MPN/g)	150(23,930)	430(43,2300)	93(9.3,430)	4.3(3.1,22.5)	23(9.3,75)
*trh* (MPN/g)	4.3(0.9,23)	4.3(0.7,23)	4.3(0.9,24)	4.3(2.3,19.0)	0.4(0.4,0.9)
*tdh* (MPN/g)	0.2(0.2,0.9)	0.2(0.2,0.9)	0.2(0.2,0.9)	0.2(0.2,0.6)	0.2(0.2,0.2)
*tdh*:*tlh* (%)	0.3(0.1,2.1)	0.2(0.0,1.0)	0.7(0.1,3.5)	4.0(1.9,8.4)	0.7(0.2,1.6)

Surface water temperature and shore water temperature were highly correlated (ρ = 0.87) with variance inflation factors in sensitivity regression models consistently reaching above 4.0, indicating that coefficient standard errors more than doubled when both variables were included in the same model. In order to avoid this collinearity, shore water temperature was excluded from multivariate regression analyses as surface water temperature provided superior model fit (ΔAIC = 121.6). Furthermore, the surface water temperature variable is more similar to water measurements used in other environmental assessments of *V. parahaemolyticus* (Duan and Su, 2005; USFDA, 2005; Paranjpye et al., 2015; Davis et al., 2017).

**FIGURE 3:**
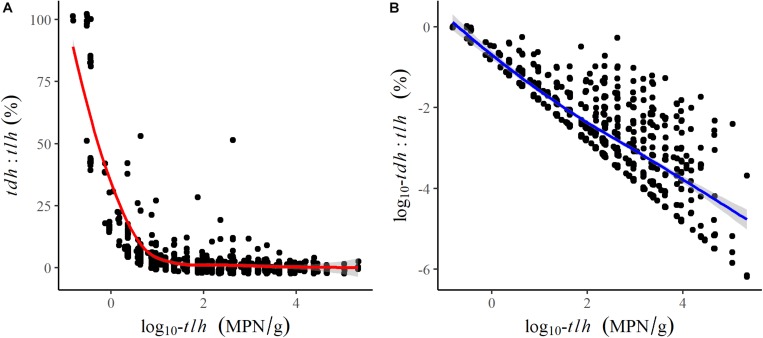
Scatter plots and fitted LOESS lines comparing the association of log-transformed *tlh* to **(A)** the *tdh*:*tlh* ratio and **(B)** the log-transformed *tdh*:*tlh* ratio.

All remaining temperature measures were well-correlated (ρ≥0.64) and were moderately positively correlated with *tlh*, *trh*, and *tdh* (Table 2). Salinity was not correlated with temperature or any genetic variables. *tlh*, *trh*, and *tdh* were positively correlated with one another, with *trh* displaying the strongest correlations. All temperature measures and *trh* were slightly negatively correlated with the *tdh*:*tlh* ratio, while *tlh* was strongly negatively correlated with the ratio, indicating that the highest relative abundance of *tdh*+ strains occurred when total abundance was close to zero. Upon further investigation, this relationship was confirmed to be log-linear; the pathogenic proportion decreased exponentially as total abundance increased (Figure 3). All aforementioned correlations were statistically significant (*p* < 0.05).

**TABLE 2:** Pearson correlation matrix of all environmental and *V. parahaemolyticus* genetic variables.

	**Ambient air**	**Surface water**	**Tissue**	**Salinity (‰)**	**Log_10_-*tlh***	**Log_10_-*trh***	**Log_10_-*tdh***	**Log_10_-*tdh*:*tlh***
	**temperature (°C)**	**temperature (°C)**	**temperature (°C)**		**(MPN/g)**	**(MPN/g)**	**(MPN/g)**	**(%)**
Ambient air temperature (°C)	–	–	–	–	–	–	–	–
Surface water temperature (°C)	0.64	–	–	–	–	–	–	–
Tissue temperature (°C)	0.83	0.68	–	–	–	–	–	–
Salinity (‰)	–0.08	–0.12	–0.12	–	–	–	–	–
Log_10_-*tlh* (MPN/g)	0.38	0.51	0.38	–0.11	–	–	–	–
Log_10_-*trh* (MPN/g)	0.44	0.46	0.45	–0.05	0.68	–	–	–
Log_10_-*tdh* (MPN/g)	0.35	0.28	0.33	–0.05	0.45	0.60	–	–
Log_10_-*tdh*:*tlh* (%)	–0.19	–0.38	–0.21	0.09	–0.82	–0.37	0.15	–

**FIGURE 4:**
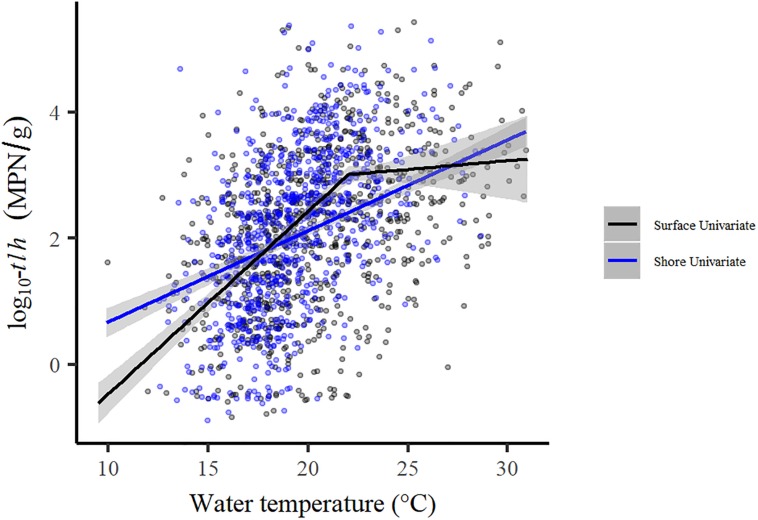
Comparison of univariate regressions for shore versus surface water temperature and total *V. parahaemolyticus*.

Tobit regression model results for total *V. parahaemolyticus* abundance (*tlh*) and strains carrying the two pathogenic markers (*trh*, *tdh*) are reported in Table 3. For all three genetic markers, each temperature variable displayed a statistically significant positive association in the univariate models. LOESS exploratory analysis identified a non-linear relationship with surface water temperature such that a strong positive association was observed up to 22°C and after which no relationship was observed (Figure 4). This non-linear threshold also improved model fit in regression models (ΔAIC = 30.2). The positive association with surface temperature was slightly attenuated for *trh* when compared to *tlh* and moderately attenuated for *tdh*. All temperature associations became attenuated in the multivariate models for *tlh*, *trh*, and *tdh*. In the multivariate *tlh* model, tissue and air temperature were no longer significantly associated with total abundance while the positive association with water temperature was only slightly attenuated, still indicating almost a doubling in total abundance for every 1°C increase up to 22°C while holding all other environmental covariates constant. For the *trh* and *tdh* multivariate models adjusting only for environmental covariates (Multivariate 1), all three temperature measures had attenuated but still statistically significant associations. For the *trh* model further adjusting for *tlh* and *tdh* (Multivariate 2), only tissue temperature maintained a statistically significant association, indicating that on average *trh* abundance approximately doubled for every 12°C increase in tissue temperature when holding all other covariates constant.

**TABLE 3:** Univariate and multivariate Tobit regression models of *tlh*, *trh*, and *tdh* and environmental/genetic covariates.

	***tlh***		***trh***			***tdh***		
**Covariate**	**Univariate**	**Multivariate**	**Univariate**	**Multivariate 1**	**Multivariate 2**	**Univariate**	**Multivariate 1**	**Multivariate 2**
Ambient air	1.27 (1.22, 1.32)	1.06 (0.99, 1.13)	1.28 (1.24, 1.32)	1.08 (1.01, 1.14)	1.01 (0.97, 1.05)	1.25 (1.19, 1.30)	1.12 (1.03, 1.21)	1.04 (0.99, 1.10)
temperature (°C)								
Surface water								
temperature (°C)								
12.5–22	1.98 (1.81, 2.16)	1.91 (1.73, 2.10)	1.81 (1.66, 1.97)	1.52 (1.38, 1.67)	1.05 (0.97, 1.13)	1.53 (1.36, 1.72)	1.27 (1.12, 1.45)	0.92 (0.83, 1.07)
22–35.4	1.02 (0.84, 1.23)	1.00 (0.83, 1.21)	0.92 (0.77, 1.11)	0.92 (0.77, 1.11)	0.92 (0.81, 1.05)	0.99 (0.78, 1.25)	1.01 (0.78, 1.30)	1.00 (0.79, 1.26)
Tissue	1.22 (1.18, 1.26)	0.96 (0.91, 1.02)	1.23 (1.20, 1.27)	1.05 (1.01, 1.10)	1.06 (1.02, 1.10)	1.19 (1.15, 1.24)	1.04 (1.01, 1.07)	1.05 (1.01, 1.10)
temperature (°C)								
Salinity (‰)	0.95 (0.91, 0.98)	1.03 (0.99, 1.06)	0.98 (0.95, 0.99)	0.99 (0.97, 1.03)	0.98 (0.96, 1.00)	0.97 (0.95, 0.99)	1.00 (0.96, 1.04)	0.99 (0.96, 1.02)
Log_10_-*tlh* (MPN/g)	–	–	4.20 (3.79, 4.68)	–	3.20 (2.84, 3.60)	3.26 (2.76, 3.85)	–	3.16 (2.61, 3.84)
Log_10_-*trh* (MPN/g)	–	–	–	–	–	5.54 (4.66, 6.58)	–	–
Log_10_-*tdh* (MPN/g)	–	–	7.78 (6.43, 9.39)	–	2.79 (2.38, 3.60)	–	–	–

*Results are displayed as the relative change in the geometric mean with a corresponding 95% confidence interval. Region and year indicator variables were included in all multivariate models.*

*tlh* and *tdh* were both strongly positively associated with *trh* before and after adjustment, although the association with *tdh* became noticeably attenuated (Table 3). For the *tdh* model, further adjusting for *tlh* (Multivariate 2) left only the tissue temperature association statistically significant, mirroring the relationship of tissue temperature with *trh*. While *tlh* maintained a strong positive association with *tdh* in the univariate and multivariate models, including *trh* as a covariate resulted in both tissue temperature and *tlh* no longer displaying statistically significant associations while *trh* maintained a particularly strong association (*e*^β^ = 3.86, 95% CI: [3.33, 4.51]). While salinity displayed a slight but statistically significant negative association in each of the univariate models for *tlh*, *trh*, and *tdh*, salinity no longer displayed a significant association with the three genetic markers in any of the multivariate models.

Each of the three genetic marker models displayed a positive statistical interaction between air and tissue temperature. The interaction suggests that at relatively low temperatures of both media no relationship between either air or tissue temperature and any genetic marker was observed, but at high temperatures of both media at least one of these temperature measures (air or tissue) had a statistically significant positive association with each genetic marker. A representative example of this interaction is shown in Table 4. When adjusting for environmental covariates, the association with surface water temperature was unaffected by this interaction. However, the slopes of air and tissue temperature were negatively transposed due to this adjustment, suggesting a negative slope at overall lower temperatures of both air and tissue (data not shown). These negative relationships should not be interpreted directly, however, as the Tobit model forces this relationship to keep the positive interaction between air and tissue temperature while also accounting for slope attenuation in both temperature measures due to the overwhelming effect of water temperature. An additional positive interaction for the *tlh* model was independently observed between surface water temperature and salinity such that the positive slope of water temperature in cooler waters (< 22°C) was steeper in more saline waters (Table 5).

**TABLE 4:** Representative example of interaction (effect modification) between tissue temperature and air temperature on genetic marker abundance.

**Covariate**	**Below median**	**Above median**
Ambient air temperature (°C)	0.91 (0.78, 1.06)	1.12 (1.02, 1.22)
Tissue temperature (°C)	1.09 (0.95, 1.25)	1.15 (1.05, 1.25)

*Relative change in geometric mean with corresponding 95% confidence intervals for *trh*, subsetting data based on whether temperatures are above or below their respective overall medians.*

**TABLE 5:** Relative change in geometric mean with corresponding 95% confidence intervals of surface water temperature (<22°C) conditioned on water salinity.

**Conditional covariate**	***tlh***	***tdh:tlh***
Surface water temperature (°C) | 3‰	1.48 (1.18, 1.86)	0.85 (0.70, 0.99)
Surface water temperature (°C) | 35‰	2.16 (1.87, 2.51)	0.59 (0.54, 0.67)

*Reported associations are adjusted for all environmental and genetic covariates as well as region and year.*

Residuals from the SUR model for *tlh* and *tdh* did not appear to be correlated (ρ = 0.05; Breusch–Pagan χ^2^ = 2.02, *p* = 0.16). Therefore, all *tdh*:*tlh* models were estimated using linear combinations of seemingly unrelated estimations of the two Tobit models (Table 6). For the pathogenic ratio, all three temperature measures had statistically significant negative associations, while salinity did not have a statistically significant association. Surface water temperature also displayed a similar non-linear relationship with this ratio, with no significant trend observed at temperatures above 22°C. After adjusting for environmental covariates (Multivariate 1) only water temperature maintained a statistically significant negative association, indicating that on average the *tdh*:*tlh* ratio approximately halved for every 2.0°C increase in water temperature up to 22°C. Tissue temperature displayed a statistically significant positive association with the pathogenic ratio, similar to the adjusted relationships observed when modeling *trh* and *tdh* concentrations but still noticeably more attenuated than the negative association with water temperature. Further adjusting for *trh* (Multivariate 2) did not alter the associations for either water or tissue temperature. In contrast to the previous multivariate models, salinity did appear to have a statistically significant negative association with *tdh*:*tlh* in the fully adjusted model. The association, however, was very slight: on average approximately halving the ratio given a 23% increase in salinity. *trh* displayed a negative association with the ratio with and without adjusting for environmental measures, although this association was much more attenuated than the other aforementioned genetic marker associations. An inverted form of the interaction between surface water temperature and salinity observed in the *tlh* models was seen for the *tdh*:*tlh* models, such that the negative slope of water temperature became steeper in more saline waters (Table 5).

**TABLE 6:** Univariate and multivariate associations between the *tdh*:*tlh* ratio and environmental/genetic covariates based on linear combinations of seemingly unrelated estimations.

	***tdh:tlh***		
**Covariate**	**Univariate**	**Multivariate 1**	**Multivariate 2**
Ambient air	0.94 (0.89, 0.99)	1.05 (0.97, 1.14)	1.04 (0.97, 1.13)
temperature (°C)			
Surface water			
temperature (°C)			
12.5–22	0.79 (0.71, 0.88)	0.67 (0.59, 0.77)	0.66 (0.58, 0.75)
22–35.4	0.97 (0.78, 1.21)	0.99 (0.77, 1.28)	0.98 (0.77, 1.24)
Tissue temperature (°C)	0.95 (0.91, 0.99)	1.07 (1.01, 1.15)	1.06 (1.01, 1.15)
Salinity (‰)	1.02 (0.99, 1.06)	0.96 (0.93, 1.00)	0.97 (0.94, 1.00)
Log10-*trh* (MPN/g)	0.71 (0.56, 0.88)	–	0.85 (0.72, 0.98)

*Results are displayed as the relative change in the geometric mean with a corresponding 95% confidence interval. Region and year indicator variables were included in both multivariate models.*

The sensitivity analysis removing samples taken in Henderson Inlet produced no substantial changes in any of the reported results. When directly compared, the univariate shore water model for *tlh* had a much more gradual slope than the surface model (*e*^β^ = 1.40, 95% CI: [1.33, 1.47]), but total abundance became more similar at higher water temperatures, primarily due to the non-linear relationship identified in the surface water model (Figure 4). Additional exploratory analysis revealed that the exponential decrease of *tdh*:*tlh* with increasing total *V. parahaemolyticus* was moderately attenuated in warmer waters (results not shown). Year and region indicator variables remained statistically significant in all multivariate regression models, consistently displaying substantial variation in total or potentially pathogenic strain abundance across space and time (results not shown). These significant trends indicate that the environmental and genetic variables considered in this study did not sufficiently explain spatial and temporal variation of each genetic marker.

## Discussion

The current analysis assessed the relationships between environmental measures and genetic markers of *V. parahaemolyticus* across space and time in a large number of Pacific oyster samples in Washington State. This study confirmed frequently reported positive associations both between temperature and absolute genetic markers as well as among the genetic markers, while also highlighting a previously unobserved upper threshold in the positive relationship with water temperature and genetic markers. This study has also confirmed strong negative associations between the *tdh*:*tlh* pathogenic ratio and both total abundance and water temperature, suggesting that an ecological relationship of competition between *V. parahaemolyticus* strains exists within the oyster tissue matrix. As such, increased attention should be given to the pathogenic ratio in future research.

Temperature, in some medium, was found to be consistently positively associated with *tlh*, *trh*, and *tdh* even after adjustment for similar environmental covariates or other genetic markers. These findings suggest that temperature is at least a moderately strong determinant of total and pathogenic *V. parahaemolyticus* abundance in oysters, independent of its impact on total abundance or the concentration of another pathogenic marker. Surface water temperature displayed the strongest relationship for all three genetic markers, however, the effect observed for *tdh* and *trh* was largely mediated by total *V. parahaemolyticus* abundance. The non-linear relationship with surface water temperature, such that no association is observed in waters warmer than 22°C, is an important addition to the understanding of ecological relationships for *V. parahaemolyticus* in PNW. While previous research has frequently shown a consistent positive relationship with water temperature in most bodies of water (Johnson, 2015), prior work in Washington State did not find that highest temperatures correlated with greatest *V. parahaemolyticus* abundance (Paranjpye et al., 2015). Current findings support this previous work by identifying a threshold relationship, such that increasing temperature beyond sufficiently warm waters (∼22°C) does not result in an increase in average total bacterium abundance. The difference in estimated abundance based on surface versus shore water temperature is also noteworthy. While the two variables are collinear, surface water temperature provides superior model fit and so appears to be the preferred environmental measure for estimating total and pathogenic *V. parahaemolyticus* abundance. However, shore water temperature was measured more frequently, likely due to the relative ease of sampling compared to surface water temperature, especially at low tide. While shore water can be used as a suitable replacement to surface water temperature when necessary, it is recommended that surface water temperature be collected during *V. parahaemolyticus* monitoring when possible.

The interaction between air and tissue temperature for all three genetic markers implies that these associations between temperature and *V. parahaemolyticus* occur primarily in sufficiently warm conditions. In other words, the highest overall abundance of *tlh* and *tdh* will likely be found when air and tissue temperatures are both near their maxima, but the lowest overall abundance will not necessarily be found at their minima. The relationship between air, water, and tissue temperature is somewhat complex given the relatively higher heat capacity of water and the strong semidiurnal tides in PNW. When oysters are exposed to ambient air during the intertidal period, there is an opportunity for oyster tissue temperatures to rise considerably and for *V. parahaemolyticus* to flourish (Jones et al., 2016). While distances between sampling sites and shifts in tidal windows did not always ensure oysters used in this study were collected soon after the tide receded, the results indicate that the deviation between tissue and water temperature across samples was mostly negligible. Bacterium associations with tissue temperature would likely strengthen if samples were consistently taken later in the intertidal period. Regardless, these findings indicate that the highest average levels of abundance for many *V. parahaemolyticus* strains occur when multiple media have higher than average temperatures.

Despite moderate variation in environmental measures, there was little to no association in the current analysis between water salinity and *tlh*, *trh*, or *tdh*. This finding is consistent with previous research in PNW (Duan and Su, 2005; Paranjpye et al., 2015). The Salish Sea and Washington coastal bays are relatively saline and none of the harvesting waters sampled in this study approached freshwater levels. The unadjusted negative associations, primarily due to lower abundance at sampling sites and time periods with waters above 30‰, became non-significant after accounting for site temperatures. This is likely due to the fact that oceanic water entering into these estuarine systems is often cooler than freshwater and therefore less hospitable to *V. parahaemolyticus* bacteria (Davis et al., 2017). The statistical interaction identified for both *tlh* and *tdh*:*tlh* indicates that salinity modifies the relationship between total *V. parahaemolyticus* abundance and surface water temperature, such that more saline waters enable warmer waters to host higher overall bacterium abundance. This finding is consistent with the consensus that *V. parahaemolyticus* thrives in environments that are both warm and saline (Naughton et al., 2009; Davis et al., 2017).

A limitation of this study is that the implemented MPN-PCR analysis is only able to quantify the overall abundance of each targeted hemolysin gene in oyster tissue samples rather than specific strains of *V. parahaemolyticus*. The results also include samples that had late amplification (i.e., real-time PCR results above 40 cycles) and therefore may have been non-specific. Although enumeration was performed via the serial dilution test, non-specific amplification results may overestimate abundance of genetic markers. However, the occurrence of late amplification was uncommon, and therefore the impact of such measurement error on the reported findings is expected to be minimal.

An additional limitation of the current analysis is that *trh*-carrying *V. alginolyticus* strains may be common in PNW waters and therefore cast doubt upon the gene’s utility as a pathogenic marker of *V. parahaemolyticus* in the region (Gonzalez-Escalona et al., 2006). While *Vibrio* spp. are often correlated in environment isolates, the strong association between the two pathogenic markers in this study could also indicate the presence of *tdh*+ /*trh*+ strains of *V. parahaemolyticus*, or alternatively both *tdh*+ /*trh*- and *tdh*-/*trh*+ strains, rather than substantial abundance of *trh*+ *V. alginolyticus*. Nevertheless, it should be noted that there are certain WDOH survey sites where samples frequently result in enumerated *trh* but not *tlh*, indicating the presence of only *V. alginolyticus* (Washington Public Health Laboratory, Personal Communication). While the *trh* concentration in these instances is often low, the potential impact of *V. alginolyticus* contributions to reported *trh* values in other samples could be substantial and therefore must be further investigated. Additional microbial analysis, such as whole-genome sequencing, would be needed to accurately determine the extent of total *trh* abundance originating from *V. alginolyticus* in PNW oyster samples. However, regardless of its source, the strong association between *tdh* and *trh*, such that its inclusion negated the relationship between *tdh* and *tlh*, suggests that the presence of the *tdh* gene is more dependent on the level of *trh* than the total abundance of *V. parahaemolyticus* in the environment.

The exponential relationship between the *tdh*:*tlh* pathogenic ratio and total abundance of *V. parahaemolyticus*, as well as its negative association with water temperature, confirm previous findings observed in Eastern oysters (*Crassostrea virginica*) in Alabama (DePaola et al., 2003). The results of the current study, which includes a larger sample size, expand upon this original finding by showing that the log-linear relationship is systematic and does not vary by sampling region or year. The consistency of these relationships in a different oyster species, decade, and region of North America suggests that they may be ubiquitous throughout the environment. Given the statistical redundancy of including the *tlh* gene in a regression model of *tdh*:*tlh*, especially for the seemingly unrelated estimation technique used in this analysis, it was impossible to formally evaluate if the negative association between water temperature and the ratio is fully explained by the relationship between temperature and total *V. parahaemolyticus* abundance. Regardless, these findings suggest that *tdh*+ strains of *V. parahaemolyticus* are more likely to be prominent in the total bacterium community in relatively cooler waters. The Food and Drug Administration’s risk model assumed that the ratio between pathogenic and total *V. parahaemolyticus* in oysters was temperature independent (USFDA, 2005). Therefore, an update to the model to account for temperature-dependent pathogenic ratio variation may improve risk predictions.

Certain strains of *V. parahaemolyticus* endemic to PNW that contain the *tdh* marker have been found to be more resilient to competitive environmental conditions (Matz et al., 2011; Nilsson et al., 2019). These findings suggest that cooler waters may provide an opportunity for *tdh*+ strains of *V. parahaemolyticus* to better establish itself among the bacterial community. The general resilience of these strains to cooler waters is of great interest, as most *V. parahaemolyticus* harvesting regulations assume that warmer temperatures result in greater risk of harvested oysters (USFDA, 2017). While previous work has identified only a marginal correlation between illness rates and *tdh*+ concentrations (Paranjpye et al., 2012), little attention has been given to the relative proportion of these strains in the environment. It may be that lower total *V. parahaemolyticus* abundance and/or cooler water temperatures provide a unique opportunity for pathogenic strains to cause infections in oyster consumers.

The marginal positive association between tissue temperature and the *tdh*:*tlh* pathogenic ratio further suggests that sudden changes in temperature may allow pathogenic strains the opportunity to establish themselves in the *V. parahaemolyticus* community. Washington State waters are generally cool given their distance from the equator. However, due to recent changes in ocean circulation, warmer waters more frequently enter into the region’s estuarine systems, which can allow *Vibrio* spp. to flourish (Martinez-Urtaza et al., 2010). A similar pattern may occur on a smaller time-scale with regard to the semidiurnal tides in PNW, where sudden warming of the surfaced oysters allows *tdh*+ strains to increase proportionately. While comparisons of genetic markers in oysters during high and low tide have been conducted (Jones et al., 2016), the study did not specifically assess the ratio of *tdh* to total abundance within samples. A similar study or re-analysis of the generated data is therefore suggested.

The moderate negative association between the *tdh*:*tlh* pathogenic ratio and total *trh* indicates that when *tdh*+ strains make up a high proportion of the local bacterium community, they may be more likely to be *tdh*+ /*trh*- strains. These strains are relatively common in the PNW environment, particularly the pandemic O3:K6 strain, although its role in vibriosis cases from consuming PNW oysters is unclear (Paranjpye et al., 2012; Turner et al., 2013; Nilsson et al., 2019). Future research evaluating the association between *tdh*:*tlh* and reported vibriosis cases in the region may help determine its utility as an indicator of the risk of foodborne illness. Such findings could also be relevant for other parts of North America given the spread of PNW strains in recent years (Martinez-Urtaza et al., 2013; Xu et al., 2017).

Seemingly unrelated estimations, while commonly applied in other scientific fields (Fiebig, 2001), has, to the authors’ knowledge, not previously been used in microbial research. Its implementation in this work allowed the regression analysis to preserve the censored values of both the numerator and denominator of the *tdh*:*tlh* ratio resulting from the limits of detection of the MPN-PCR technique. While most of the censored data from the pathogenic ratio were due to the high proportion of *tdh* measurements below the limit of detection, exploratory statistical analysis indicated that the corresponding values for *tlh* varied across the entire range of observable data. A more simplified approach, such as treating all aforementioned ratio measures as left-censored, would therefore have introduced bias. The seemingly unrelated estimation of the separate Tobit models was a preferable alternative, allowing for the linear relationships between the latent Tobit variables and regression covariates to be simultaneously compared for the numerator and denominator of the ratio. The technique applied in this work is unbiased when the residuals of the two regressions are uncorrelated. If this had not been the case, a more complex approach, such as SUR for Tobit regressions (Arouna and Dabbert, 2010), would have been more appropriate. Seemingly unrelated statistical techniques should be considered in future microbial research that examines ratios of different genetic markers, particularly when data are censored.

The simple imputation for censored genetic data implemented in this work is likely why the log-transformed *tdh* variable appeared positively skewed and in general may have biased reported correlations and regression associations toward the null. This statistical bias is unlikely to impact total *V. parahaemolyticus* abundance given the low rate of censoring for *tlh*. However, *trh* and particularly *tdh* may be prone to this bias. While a stochastic imputation following the lognormal distributions of the genetic variables could have been implemented as an alternative approach (Wei et al., 2018), this statistical technique seemed unnecessary as the only non-significant relationship between genetic markers was for the *tlh* covariate in the *tdh* model after adjusting for *trh*. Therefore, such an imputation would not be expected to substantially alter the results of the current analysis.

The environmental findings reported in this study arise from one of the largest samples of oysters enumerated for *V. parahaemolyticus* ever collected. To the authors’ knowledge, it is the largest dataset of its kind available for the PNW and covers a wide array of growing areas in Washington State. The strength of the reported associations and the lack of effect modification by harvesting region increase the likelihood that these findings are generalizable to Washington State and likely other shellfish harvesting waters in the PNW. However, it is also likely that the temperature and salinity associations with the genetic markers reported here are unique to this region of North America, as other research has shown consistently linear positive relationships with water temperature and complex non-linear relationships with salinity in other bodies of water (DePaola et al., 2003; USFDA, 2005; Johnson, 2015; Davis et al., 2017). It is recommended that similar analyses be conducted on different oyster samples in PNW to evaluate the consistency of the current findings across this region.

While the associations reported in this work are noteworthy, additional environmental information, such as water clarity and quality (e.g., turbidity, oxygenation, presence of nutrients, plankton abundance) as well as watershed precipitation and land use, was not readily available for analysis. This missing information may explain some of the associations observed in this work, particularly the residual spatial and temporal variation in the models (Paranjpye et al., 2015; Davis et al., 2017; Nilsson et al., 2019). Therefore, the current results from the multivariate models should be interpreted with caution. Future work can address this limitation by drawing from additional environmental datasets in Washington State and using spatial–temporal statistical techniques to predict these environmental measures at the same place and time as oyster sampling. Similar statistical techniques could also be used to account for potential similarities across oyster samples based upon time of year and specific sampling coordinates. Characterizing these space–time statistical dependencies in a regression framework may further modify the associations observed in this work. These aforementioned models will be utilized in future work.

## Conclusion

Abundance of total *V. parahaemolyticus* and potentially pathogenic strains in Pacific oysters from Washington State were found to have clear positive associations with a variety of measured temperatures and with each other. The relative abundance of *tdh*+ strains was negatively associated with water temperature, total *V. parahaemolyticus*, and *trh*+ strains. This work further characterizes the environmental determinants of the bacterium including potentially pathogenic strains in Pacific oysters. The inclusion of tissue and air temperature, as well as accounting for the non-linear trend in surface water temperature, will also likely improve *V. parahaemolyticus* estimations used for shellfish safety risk management. These findings lay the groundwork for future characterizations of the bacterium in the region that will attempt to demonstrate how pre-harvest variation relates to the risk of foodborne illness among oyster consumers.

## Data Availability Statement

The datasets generated for this study are available on request to the corresponding author.

## Author Contributions

AF implemented the data analysis and drafted the original manuscript for this work. BD mentored AF, conceived and designed the study, and edited analysis and writing as needed. EA and GO represent the Washington State Department of Health, which collected all oyster samples and environmental measures, conducted microbial analysis, and performed quality assurance and quality control on the dataset. JB assisted in the design and implementation of the regression analysis. AD assisted with study design, writing, and interpretation of the findings. FC advised all authors throughout, specifically supervising the conceptual design, data analysis, and manuscript writing.

## Conflict of Interest

AD is a retired USFDA employee, now sole proprietor consultant of Angelo DePaola Consulting. The remaining authors declare that the research was conducted in the absence of any commercial or financial relationships that could be construed as a potential conflict of interest.
